# Editorial: Climate change and cardiovascular health

**DOI:** 10.3389/fphys.2024.1497232

**Published:** 2024-10-08

**Authors:** Antonia Kaltsatou, Josh Foster, Tiina M. Ikäheimo

**Affiliations:** ^1^ FAME Laboratory, Department of Physical Education and Sport Science, University of Thessaly, Trikala, Greece; ^2^ Centre for Human and Applied Physiological Sciences, Faculty of Life Sciences and Medicine, King’s College, London, United Kingdom; ^3^ Department of Community Medicine, UiT The Arctic University of Norway, Tromsø, Norway; ^4^ Research Unit of Population Health, University of Oulu, Oulu, Finland

**Keywords:** climate change, cardiovascular health, public health, environmental stressors, heat stress, heat-related illness, extreme weather events, cold stress

## Introduction

Climate change is increasingly recognized as the most significant health threat facing humanity (Patz et al., 2014). Its effects manifest through a rise in the frequency and severity of extreme weather events, which pose a serious risk to vulnerable populations, especially those with cardiovascular diseases (CVD) (De Blois et al., 2015). As the global climate continues to change, it is crucial to understand how these environmental stressors affect individuals with pre-existing health conditions. This editorial aimed to synthesize and discuss the findings of the published studies that explore the intersection between climate change, extreme weather, and cardiovascular health, particularly focusing on populations at risk.

The burden of CVDs is exacerbated by extreme weather events, which are becoming more frequent and severe due to climate change. Heatwaves, cold spells, and fluctuating environmental conditions all pose significant threats to cardiovascular health. Individuals with compromised cardiovascular systems, such as those suffering from coronary artery disease (CAD), heart failure, hypertension, or stroke, are particularly vulnerable. The studies published in this Research Topic contribute to our understanding of how extreme temperatures and environmental factors influence cardiovascular outcomes, highlighting the urgent need for targeted preventive strategies.

One of the studies explored the association between environmental temperature, relative humidity, and cardiovascular disease among older adults in China (Chen and Zhang). Using data from the China Longitudinal Health and Longevity Survey (CLHLS), the researchers found that higher average annual temperatures and relative humidity levels were associated with a reduced risk of CVD, including hypertension, heart disease, and stroke. Specifically, a 1°C increase in temperature was linked to a decrease in the prevalence of these conditions, while a 1% increase in relative humidity also contributed to lower CVD rates, particularly at higher humidity levels. These findings underscore the complex relationship between environmental factors and cardiovascular health. While extreme cold and low humidity were independently associated with higher CVD risks, moderate increases in temperature and humidity appeared to have protective effects. This study highlights the importance of considering regional climate variations when assessing cardiovascular risk and emphasizes the need for public health strategies that account for these environmental factors.

Wildland fires are another significant environmental hazard exacerbated by climate change. In this Research Topic, a review focuses on the long-term cardiovascular impacts of exposure to wildland fire smoke, particularly the effects on the microvasculature (Naserinejad et al.). Particulate matter (PM) from wildland fires, especially PM2.5 and ultrafine particles (UFP), can penetrate deep into the respiratory system and enter the bloodstream. Once in the bloodstream, these particles can interact with the endothelium of the microvasculature, leading to dysfunction and potentially severe cardiovascular conditions.

Populations with pre-existing microvascular vulnerabilities, such as the elderly or those with cardio-metabolic diseases, are at heightened risk of adverse cardiovascular outcomes following exposure to wildland fire smoke. Despite the growing recognition of these risks, significant gaps remain in our understanding related to the mechanisms behind exposure to air pollution and cardiovascular health. The review calls for more research using microvascular endothelial cells and animal models that closely mimic human exposure to wildland fire smoke. Additionally, population-based studies are needed to provide more accurate data on PM exposure and its impact on cardiovascular health, particularly in vulnerable populations.

Another study analyzed hospitalization rates for cardiovascular diseases in Ganzhou City, Jiangxi Province, China, from 2016 to 2020 (Yan et al.). The researchers aimed to uncover the spatiotemporal distribution characteristics and influencing factors of CVD hospitalization rates. Using data analysis tools like ArcGIS, SaTScan, and Matlab, the study identified significant trends, including seasonal variations with higher hospitalization rates in winter and summer. The study also highlighted the prevalence of CVDs among individuals aged 61 and above, with significant spatiotemporal clustering in areas like Zhanggong District and Nankang City. Environmental factors such as NO_2_ and O_3_ concentrations, average annual temperature, and annual maximum temperature diurnal range were found to significantly influence CVD hospitalization rates. The study noted that sudden temperature changes had the most significant impact on cardiovascular health, emphasizing the need for targeted prevention strategies during critical periods, particularly in high-risk areas. The findings from this study provide valuable insights into the spatial and temporal distribution of CVD hospitalization rates in Ganzhou City. The study’s conclusions emphasize the importance of addressing environmental factors, such as air pollution and temperature fluctuations, in public health strategies to mitigate the impact of climate change on cardiovascular health.

A cross-over trial explored the effects of cold environments on the autonomic nervous system (ANS), baroreflex sensitivity (BRS), and blood pressure variability (BPV) during post-exercise recovery in patients with stable coronary artery disease (CAD) (Pikkarainen et al.). The study compared the cardiovascular responses of CAD patients following static and dynamic upper-body exercises performed in cold (−15°C) and neutral (+22°C) environments.

The key findings suggest that static upper-body exercise in a cold environment leads to increased post-exercise vagal activity and baroreflex sensitivity. This was evidenced by higher high-frequency (HF) spectral power of heart rate variability (HRV), reduced heart rate, and increased BRS. In contrast, dynamic upper-body exercise in a cold environment did not significantly alter post-exercise autonomic and cardiovascular responses compared to a neutral environment, except for a reduction in HF BRS. These findings are significant for planning year-round health enhancing exercise-based programs for CAD patients, especially in cold climates. The study highlights the need for further research on how different forms of exercise in varying environmental conditions impact ANS function, BRS, and BP among individuals with cardiovascular diseases. The results are particularly relevant in the context of climate change and the increasing frequency of extreme weather events.

The final study discussed in this editorial focused on validating existing formulae for estimating stroke volume (SV) in workers exposed to heat during their work shifts (Tsoutsoubi et al.). Monitoring cardiovascular strain, such as heart rate and blood pressure, is crucial for workers exposed to hot environments, where 35% experience symptoms of heat strain (Flouris et al., 2018). The study assessed the accuracy of existing equations for predicting SV, which could be useful in occupational health monitoring. In this study, SV was measured using finger photoplethysmography (Finapres) against SV calculated using various formulae from the literature. The study found that estimating stroke volume using existing formulae is feasible, with good correlation and relatively small bias, especially when workers are in a supine position under thermoneutral conditions. Although predictions were less accurate in an upright posture and hot environment, the results were still reasonable, indicating that these formulae can be used to assess cardiovascular strain in workers.

The study concludes that monitoring SV during breaks could be a practical solution for occupational health monitoring, particularly in hot conditions. This approach could help protect workers from the adverse cardiovascular effects of heat exposure, especially during heatwaves, which are becoming more frequent due to climate change.

## Conclusion

The studies discussed in this editorial provide important insights into the complex relationship between climate change, extreme weather, and cardiovascular health. They underscore the urgent need for targeted prevention and control strategies to protect vulnerable populations, particularly those with pre-existing cardiovascular conditions. As the global climate continues to change, it is crucial to advance our understanding of how environmental factors influence cardiovascular outcomes and to develop effective interventions to adapt to the environmental changes and, on the other hand, mitigate these risks.

In particular, the studies highlight the need for more research on the microvascular effects of air pollution related to wildland fires, the spatiotemporal distribution of CVD hospitalization rates, and the cardiovascular responses to cold and heat exposure. By addressing these knowledge gaps, we can better assess the health risks posed by climate change and develop targeted measures to protect public health in an era of growing environmental challenges.

Furthermore, the findings emphasize the importance of considering regional climate variations and the specific vulnerabilities of different populations when developing public health strategies. As the world continues to grapple with the effects of climate change, it is imperative that we prioritize the health and wellbeing of those most at risk, particularly individuals with cardiovascular diseases. Through continued research and the development of targeted interventions, we can work towards a future where the adverse health impacts of climate change are effectively reduced and where all individuals can live healthy, resilient lives.
